# A new species of the genus *Lycodon* (Serpentes, Colubridae) from Guangxi, China

**DOI:** 10.3897/zookeys.954.53432

**Published:** 2020-07-29

**Authors:** Jian Wang, Shuo Qi, Zhi-Tong Lyu, Zhao-Chi Zeng, Ying-Yong Wang

**Affiliations:** 1 State Key Laboratory of Biocontrol/The Museum of Biology, School of Life Sciences, Sun Yat-sen University, Guangzhou 510275, China Sun Yat-sen University Guangzhou China; 2 School of Ecology, Sun Yat-sen University, Guangzhou 510006, China Sun Yat-sen University Guangzhou China

**Keywords:** Colubrinae, Guangxi, *Lycodon cathaya* sp. nov., morphology, phylogeny, taxonomy

## Abstract

A new species of colubrid snake, *Lycodon cathaya***sp. nov.**, is described based on two adult male specimens collected from Huaping Nature Reserve, Guangxi, southern China. In a phylogenetic analyses, the new species is shown to be a sister taxon to the clade composed of *L. futsingensis* and *L. namdongensis* with low statistical support, and can be distinguished from all known congeners by the significant genetic divergence in the mitochondrial cytochrome *b* gene fragment (*p*-distance ≥ 7.9%), and morphologically by the following combination of characters: (1) dorsal scales in 17–17–15 rows, smooth throughout; (2) supralabials eight, third to fifth in contact with eye, infralabials nine; (3) ventral scales 199–200 (plus two preventral scales), subcaudals 78; (4) loreal single, elongated, in contact with eye or not, not in contact with internasals; (5) a single preocular not in contact with frontal, supraocular in contact with prefrontal, two postoculars; (6) maxillary teeth 10 (4+2+2+2); (7) two anterior temporals, three posterior temporals; (8) precloacal plate entire; (9) ground color from head to tail brownish black, with 31–35 dusty rose bands on body trunk, 13–16 on tail; (10) bands in 1–2 vertebral scales broad in minimum width; (11) bands separate ground color into brownish black ellipse patches arranged in a row along the top of body and tail; (12) elliptical patches in 3–6 scales of the vertebral row in maximum width; (13) ventral surface of body with wide brownish black strip, margined with a pair of continuous narrow greyish white ventrolateral lines. With the description of the new species, 64 congeners are currently known in the genus *Lycodon*, with 16 species occurring in China.

## Introduction

The colubrid genus *Lycodon* Boie, 1827 currently comprises 63 known species, and is distributed widely throughout the Middle East to Southeast Asia, as well as to the Indo-Australian Archipelago (Lanza 1999; Siler et al. 2013; Neang et al. 2014; Uetz et al. 2020). Fifteen species have so far been recorded from China, i.e. *L. aulicus* (Linnaeus, 1758), *L. fasciatus* (Anderson, 1879), *L. flavozonatus* (Pope, 1928a), *L. futsingensis* (Pope, 1928b), *L. gongshan* Vogel & Luo, 2011, *L. laoensis* Günther, 1864, *L. liuchengchaoi* Zhang, Jiang, Vogel & Rao, 2011, *L. meridionalis* Bourret, 1935, *L. multizonatus* (Zhao & Jiang, 1981), *L. rosozonatus* (Hu & Zhao, 1972), *L. rufozonatus* Cantor, 1842, *L. ruhstrati* (Fischer, 1886), *L. septentrionalis* (Günther, 1875), *L. subcinctus* Boie, 1827 and *L. synaptor* Vogel & David, 2010 (Zhao. 1981; Zhao et al. 1998; Luo et al. 2010; Vogel and David 2010; Vogel and Luo 2011; Zhang et al. 2011).

**Figure 1.**
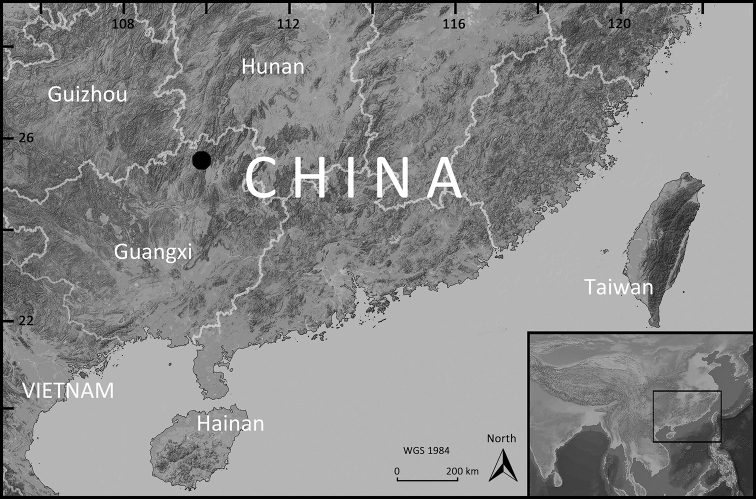
The type locality of *Lycodon cathaya* sp. nov., Huaping Nature Reserve, Guangxi, China.

During recent herpetological surveys in Guangxi, southern China, two colubrid snake specimens were collected from Huaping Nature Reserve (Fig. 1). Detailed morphological examinations and further molecular analyses revealed that these specimens represented a separately evolving lineage within the genus *Lycodon* and can be distinguished from all recognized congeners. We herein describe this overlooked *Lycodon* population as a new species, based on an integrative taxonomic approach.

## Materials and methods

### Morphometrics

Morphological examinations were performed based on two specimens collected from Huaping Nature Reserve, Guangxi, China. All specimens were fixed in 10 % buffered formalin and later transferred to 70 % ethanol for permanent preservation, and deposited in the Museum of Biology, Sun Yat-sen University (**SYS**).

Morphological descriptions followed Dowling (1951), Vogel (2009), Vogel and David (2010), and Janssen et al. (2019). Measurements were taken with digital calipers to the nearest 0.1 mm. These measurements were as follows:

**ED** eye horizontal diameter;

**HL** head length (from tip of snout to posterior margin of the mandible);

**HW** maximum head width;

**SVL** snout-vent length (from tip of snout to posterior margin of cloacal plate);

**TaL** tail length (from posterior margin of cloacal plate to tip of tail);

**TL** total length (from tip of snout to tip of tail).

Scalation features and their abbreviations are as follows: dorsal scale rows (**DSR**) counted at one head length behind head, at midbody, and at one head length before vent, respectively; supralabials (**SPL**); numbers of supralabials in contact with the eye (**SPL-E**); infralabials (**IFL**); chin shields (**CS**); numbers of infralabials in contact with the anterior chin shield (**IFL-aCS**); number of infralabials in contact with the posterior chin shield (**IFL-pCS**); preoculars (**PrO**); postoculars (**PtO**); loreal (**LoR**); loreal in contact with the eye or not **(L-E)**; anterior temporals (**aTMP**); posterior temporals (**pTMP**); preventral scales (**PrV**); ventral scales (**V**); precloacal plate (**PrC**); subcaudals (**SC**); and body scale surface (**BSC**). Sex was determined by dissection or by the presence/absence of everted hemipenis. The number of maxillary teeth (**MT**) were counted by carefully dissecting the gums of the right maxilla under the stereo microscope. The light bands on the body and tail were counted on one side; hardly visible or incomplete bands were counted as one band; obviously fused bands were counted as two bands. The collar band on the neck was not included in counts and bands covering the cloacal plate were regarded as body bands.

Morphological characters of recognized *Lycodon* species were obtained from examination of museum specimens (see Appendix 1) and from the following references: Günther (1864), Günther (1875), Blanford (1878), Boulenger (1893), Boulenger (1900), Wall (1906), Stejneger (1907), Griffin (1909), Taylor (1922), Pope (1928a, b), Smith (1943), Taylor (1950), Leviton (1965), Hu et al. (1975), Zhao (1981), Ota and Ross (1994), Manthey and Grossman (1997), Captain (1999), Lanza (1999), Slowinski et al. (2001), Daltry and Wüster (2002), Gaulke (2002), Gaulke et al. (2003), Jackson and Fritts (2004), Vijayakumar and David (2005), Zhao (2006), Mukherjee and Bhupathy (2007), Mistry et al. (2007), Vogel et al. (2009), Bahuguna and Bhuta (2010), Vogel and David (2010), Vogel and Luo (2011), Zhang et al. (2011), Vogel et al. (2012), Guo et al. (2013), Vogel and Harikrishnan (2013), Grismer et al. (2014), Lei et al. (2014), Neang et al. (2014), Zhang et al. (2015), Gawor et al. (2016), Do et al. (2017), Wostl et al. (2017), Ganesh and Vogel (2018), Luu et al. (2018), Melvinselvan et al. (2018), Janssen et al. (2019), Luu et al. (2019), and Vogel and David (2019). Data shown in Table 1 was modified based on Janssen et al. (2019), with distinguishing characters marked in bold.

**Table 1.** Selected morphological characters of *Lycodon* species for comparison (after Janssen et al. 2019, see Materials and methods). Bold font indicates distinguishing characteristics.

***Lycodon* species**	***cathaya* sp. nov.**	*** albofuscus ***	*** alcalai ***	*** anamallensis ***	*** aulicus ***	*** banksi ***	*** bibonius ***	*** butleri ***	*** capucinus ***	*** cardamomensis ***	*** carinatus ***
**DSR**	17–17–15	?–17–?	**19**–17–15	17–17–15	17–17–15	17–17–15	**19**–17–15	?–17/19–?	17–17–15	**19**–17–15	17/19–**19**–17
**MT**	10	**12**	**11**–**13**	?	?	?	**11**–**14**	?	**15**	10–12	?
**SPL**	8	8	**9**	**9**	8–10	8	7–9	8–9	**9**–**10**	**9**–**10**	8–9
**SPL-E**	3^rd^–5^th^	3^rd^–5^th^	**4^th^**–**5^th^**	3^rd^–5^th^	3^rd^–5^th^	3^rd^–5^th^	3^rd^/4^th^–5^th^	3^rd^–5^th^	3^rd^–5^th^	3^rd^–5^th^	3^rd^–5^th^
**IFL**	9	?	**10**	**10**–**11**	**10**–**11**	**10**	9–10	9–10	9–10	9–10	?
**PrO**	1	1	**2**	1	1	1	**2**	1	1	1	1
**PtO**	2	2	**3**	2–3	2	2	2–3	2	2	2–3	2
**Loreal**	1	1	1	1–2	1	1	1	1	1	1	1
**L-E**	yes/no	no	no	?	no	no	no	yes	no	no	no
**aTMP**	2	2	2	2	2	2	2+3	2	2	2	2
**pTMP**	3	**2**	3	**3**+**4**	3	3	**2**+**3**+**3**	**2**	3	2–3	2–3
**V**	199–200	**241**	**203**–**207**	174–204	180–215	**241**	**204**–**212**	**220**–**227**	182–211	**215**–**228**	185–202
**SC**	78	**155**–**208**	**108**–**126**	**60**–**74**	57–78	26 (broken tail)	**110**–**120**	**81**–**96**	**59**–**74**	**87**–**93**	**51**–**64**
**PrC**	entire	**divided**	entire	entire /divided	**divided**	entire	entire	entire	**divided**	entire	entire
**BSC**	smooth	**keeled**	smooth	smooth	smooth and glossy	smooth (**six central DSR of posterior 1/3 feebly keeled**)	smooth	**keeled**	**weakly keeled**	**weakly keeled**	**strongly keeled**
***Lycodon* species**	*** cavernicolus ***	*** chrysoprateros ***	*** davidi ***	*** davisonii ***	*** dumerilii ***	*** effraenis ***	*** fasciatus ***	*** fausti ***	*** ferroni ***	*** flavicollis ***	*** flavomaculatus ***
**DSR**	17–17–15	**19**–17–15	17–17–15	?–13–?	**19**–17–15	?–17–?	17–17–15	**19**–17–15	**19**–17–15	17–17–15	17–17–15
**MT**	?	**11**–**13**	**11**	?	**13**–**15**	?	**11**	**13**	**12**	?	?
**SPL**	**9**–**10**	**9**	8	**7**	**11**–**13**	**9**	8	**9**	**10**	**9**	**9**
**SPL-E**	**4^th^**–**6^th^**	3^rd^–5^th^	3^rd^–5^th^	**3^rd^**–**4^th^**	**4^th^**–**5^th^**	3^rd^–5^th^	3^rd^–5^th^	**4^th^**–**5^th^**	**4^th^**–**6^th^**	3^rd^–5^th^	3^rd^–5^th^
**IFL**	**10**–**11**	**10**	**10**	**8**	9–10	**10**–**11**	8–10	9–10	**10**	**11**	**10**
**PrO**	1	**2**	1	1	1–2	1	1	**2**	**2**	1	1
**PtO**	2	2–3	2	2–3	2	2–3	2	**3**	2	2	2
**Loreal**	1	1	1	1	1	**0**	1	1	1	1	1
**L-E**	yes	no	no	yes	yes/no	**no LoR**	yes	?	no	no	no
**aTMP**	2–3	**2**+**3**+**4**	2	1–2	2	2	2	2	2	2–3	1–2
**pTMP**	3–4	**2**+**3**+**4**	2–3	**2**	3	2–3	**2**	2–3	**3**+**4**	3 (rarely 2)	3 (rarely 2)
**V**	**232**–**245**	**186**–**194**	**224**	**233**–**265**	195–221	**215**–**228**	182–225	**207**–**215**	**203**	**210**–**224**	**165**–**183**
**SC**	**92**–**113**	**111**–**117**	**99**	**90**–**108**	**111**–**120**	72–99	65–94	**135**–**148**	**109**	**65**–**72**	**53**–**63**
**PrC**	entire	entire	entire	entire	entire	entire	entire	entire	entire	**divided**	**divided**
**BSC**	**the 8 medial rows weakly keeled**	smooth	**middorsal scale rows slightly keeled**	smooth	?	smooth	**keeled**	smooth	smooth	smooth with single apical pit	smooth
***Lycodon* species**	*** flavozonatus ***	*** futsingensis ***	*** gammiei ***	*** gibsonae ***	*** gongshan ***	*** gracilis ***	*** hypsirhinoides ***	*** jara ***	*** kundui ***	*** laoensis ***	*** liuchengchaoi ***
**DSR**	17–17–15	17–16/17–15	17–17/19–15	17–17–15	17–17–15	?–**15**–?	17–17–15	17–17–15	**15**–**15**–**15**	17–17–15	17–17–15
**MT**	**13**	**12**–**15**	?	**13**	?	**9**	?	?	?	?	**8**–**9**
**SPL**	8	7–8	7–9	8	8	8	**9**	8–9	**7**	**9**–**10**	7–8
**SPL-E**	3^rd^–5^th^	2–4; 3/4–5; 4–6	3^rd^–4^th^/5^th^	3^rd^–4^th^/5^th^	3^rd^–5^th^	**3^rd^**–**^4t^**h	3^rd^–5^th^	3^rd^–5^th^	**3^rd^**–**4^th^**	3^rd^–5^th^	3^rd^–5^th^
**IFL**	**10**	9–11	?	**10**	**8**	?	**10**	?	?	**10**	7–9
**PrO**	1	1	1	1	1	**2**	1	1	?	1	1
**PtO**	2	2–3	1–2	2	2	2	2	2	2	2–3	2
**Loreal**	1	1	1	1	1	1	1	1	1	1	1
**LoR-E**	no	no	no	yes	yes	yes	no	no	no	no	yes
**aTMP**	2	1–2	2 or irregular	2	2	2	2	1–2	**1**	2	1–3
**pTMP**	2–3	2–3	2 or irregular	3	2–3	3	3	2–3	**2**	3	1–3
**V**	**211**–**221**	193–208	**205**–**220**	**223**–**226**	**210**–**216**	**234**	188–210	**167**–**188**	**186**	**163**–**192**	190–228
**SC**	**80**–**88**	72–87	**98**–**111**	**91**–**92**	**92**–**96**	**81**–**83**	**61**–**75**	**52**–**74**	**70**	**60**–**76**	**68**–**75**
**PrC**	entire /divided	entire	entire	entire	entire	entire	**divided**	**divided**	entire /divided	**divided**	**divided**
**BSC**	**the 7 medial rows feebly keeled**	smooth	**the 9 medial rows keeled**	**upper 3 or 4 rows keeled**	**the 7–13 medial rows keeled**	**keeled**	smooth	smooth	smooth	smooth	**feebly keeled in median rows**
***Lycodon* species**	*** mackinnoni ***	*** meridionalis ***	*** muelleri ***	*** multifasciatus ***	*** multizonatus ***	*** namdongensis ***	*** nympha ***	*** ophiophagus ***	*** orientalis ***	*** paucifasciatus ***	*** philippinus ***
**DSR**	17–17–15	17–17–15	**19**–17–15	17–17–?	17–17–15	17–17–15	**13**–**13**–**13**	17–17–15	?–17–?	**19**–17/19–15	?–15–?
**MT**	?	**11**	**14**–**15**	?	10–11	**12**	8–10	**11**–**13**	10–11	**11**–**12**	**8**
**SPL**	7–8	8	**9**	?	7–8	8	6–8	8	8	8	**7**
**SPL-E**	3^rd^–5^th^	3^rd^–5^th^	**4^th^**–**5^th^**	?	3^rd^–5^th^	3^rd^–5^th^	**3^rd^**–**4^th^**	3^rd^–5^th^	3^rd^–5^th^	3^rd^–5^th^	**3^rd^**–**4^th^**
**IFL**	**8**	**10**	**10**	?	**7**–**8**	**10**	?	**10**	?	**10**	**7**
**PrO**	1	1	1–2	?	0–1	1–2	1–2	1	**0**	1	0–1
**PtO**	2	2	2–3	?	2	**3**	2	2	2	2	2–3
**Loreal**	0–1	1	1	?	1	1	1	1	1	1	1
**L-E**	no	no	no	no	yes	no	yes	no	yes	no	yes
**aTMP**	1–2	2	2	?	1–2	2	2	2	2	2	2
**pTMP**	2–3	3	**3**+**4**	?	2–3	**2**	2–3	3	3	3	3
**V**	**163**–**187**	**227**–**240**	**205**–**213**	**229**–**237**	**190**–**195**	**218**	200–243	**211**–**212**	200–208	**219**–**222**	**216**–**225**
**SC**	**48**–**56**	**96**–**106**	**112**–**117**	**106**–**119**	**68**–**75**	**85**	65–88	**87**–**90**	**68**–**74**	**90**–**92**	**87**–**99**
**PrC**	**divided**	**divided**	entire	?	**divided**	entire	**divided**	entire	**divided**	entire	entire
**BSC**	smooth	**the 10–12 medial rows feebly keeled**	?	**keeled**	smooth	smooth	**keeled**	smooth	**scales with a very faint keel along their anterior half**	**the 3–5 medial rows distinctly keeled**	smooth
***Lycodon* species**	*** pictus ***	*** rosozonatus ***	*** rufozonatus ***	*** ruhstrati ruhstrati ***	***ruhstrati abditus***	*** sealei ***	*** semicarinatus ***	*** septentrionalis ***	*** sidiki ***	*** solivagus ***	*** stormi ***
**DSR**	17–17–15	**19**–**19**–15/17	17/19–17–15	17–17–15		?–17–?	?–17–?	17–17–15	17–17–15	**19**–17–15	?–**19**–?
**MT**	**13**–**14**	**12**–**13**	**11**–**13**	?	**11**–**13**	?	?	**7**	**7**	**11**–**13**	?
**SPL**	8	8	8	8		?	8	8	8	**9**	8
**SPL-E**	3^rd^–5^th^	?	3^rd^–5^th^	3^rd^–5^th^		?	3^rd^–5^th^	3^rd^–5^th^	3^rd^–5^th^	**4^th^**–**5^th^**	**3^rd^**–**4^th^**
**IFL**	**10**	?	9–10	9–10	9–11	?	?	**7**–**8**	9–10	**10**	?
**PrO**	1	1	1	1		**0**	1	1	**0**	**2**	1
**PtO**	2	2	2	1–2	2	?	2	2	2	2–3	2
**Loreal**	1	1	1	1	1	1	1	1	1	1	1
**L-E**	yes	no	no	no		yes	no	no	yes	no	no
**aTMP**	2	2	2	1–2		?	2	2	2	2	**1**
**pTMP**	3	3	3	2–3		?	3	3	**2**	3	3
**V**	**212**–**218**	**221**–**234**	184–225	**212**–**228**	197–229	?	**211**–**234**	**202**–**224**	**195**	198–203	**217**
**SC**	**90**–**91**	?	53–98	**97**–**114**	**90**–**103**	?	65–105	**83**–**104**	**85**	**112**–**115**	**75**
**PrC**	entire	?	entire	entire		**divided**	entire	entire	**divided**	entire	entire
**BSC**	smooth	**weakly keeled**	**feebly keeled in the posterior body part**	**the 7–13 medial rows distinctly keeled**	**the 5 medial rows distinctly keeled**	?	**keeled along anterior half (4 outer rows smooth)**	**the 7/9 medial rows feebly keeled**	**keeled**	smooth	smooth
***Lycodon* species**	*** striatus ***	*** subannulatus ***	*** subcinctus ***	*** striatus ***	*** subannulatus ***	*** subcinctus ***	*** synaptor ***	*** tessellatus ***	*** tiwarii ***	*** travancoricus ***	*** tristrigatus ***
**DSR**	17–17–15	**15**–**15**–**15**	17–17–15	17–17–15	**15**–**15**–**15**	17–17–15	15/17–17–15	17–17–15	?–17–15	17–17–15	?–**15**–?
**MT**	?	8–10	8–14	?	8–10	8–14	10	?	?	?	8–10
**SPL**	**9**	**7**	8	**9**	**7**	8	8	8–9	?	**9**	**7**
**SPL-E**	3^rd^–5^th^	**3^rd^**–**4^th^**	3^rd^–5^th^/6^th^	3^rd^–5^th^	**3^rd^**–**4^th^**	3^rd^–5^th^/6^th^	3^rd^–5^th^	**4^th^**–**5^th^**	?	3^rd^–5^th^	**3^rd^**–**4^th^**
**IFL**	**11**	**8**	**7**–**8**	**11**	**8**	**7**–**8**	**8**	?	?	?	?
**PrO**	1	1	**0**	1	1	**0**	1	1	?	1	**0**
**PtO**	2	2	2–3	2	2	2–3	2	2	?	2	2
**Loreal**	1	1	1	1	1	1	1	1	?	1	1
**L-E**	no	yes	yes	no	yes	yes	no	no	?	no	yes
**aTMP**	2 (rarely 1)	2	1	2 (rarely 1)	2	1	2	2	?	2–3	2
**pTMP**	3 (rarely 2)	**2**	2	3 (rarely 2)	**2**	2	**2**	2–3	?	3	2–3
**V**	**153**–**178**	**225**–**244**	190–230	**153**–**178**	**225**–**244**	190–230	**201**–**203**	**222**–**232**	**218**–**237**	176–206	**224**
**SC**	**42**–**66**	**93**–**111**	60–91	**42**–**66**	**93**–**111**	60–91	**68**–**69**	**56**	61–102	**64**–**76**	**86**
**PrC**	**divided**	entire	entire /divided	**divided**	entire	entire /divided	entire	**divided**	**divided**	entire	entire
**BSC**	smooth	**keeled**	**feebly keeled**	smooth	**keeled**	**feebly keeled**	**the 6–7 medial rows keeled**	smooth	?	smooth	**keeled**
***Lycodon* species**	*** striatus ***	*** subannulatus ***	*** subcinctus ***	*** synaptor ***	*** tessellatus ***	*** tiwarii ***	*** travancoricus ***	*** tristrigatus ***	*** zawi ***	*** zoosvictoriae ***	
**DSR**	17–17–15	**15**–**15**–**15**	17–17–15	15/17–17–15	17–17–15	?–17–15	17–17–15	?–**15**–?	17–17–15	17–17–15	
**MT**	?	8–10	8–14	10	?	?	?	8–10	**12**	**9**	
**SPL**	**9**	**7**	8	8	8–9	?	**9**	**7**	8–9	8	
**SPL-E**	3^rd^–5^th^	**3^rd^**–**4^th^**	3^rd^–5^th^/6^th^	3^rd^–5^th^	**4^th^**–**5^th^**	?	3^rd^–5^th^	**3^rd^**–**4^th^**	3^rd^–5^th^	3^rd^/4^th^–5^th^	
**IFL**	**11**	**8**	**7**–**8**	**8**	?	?	?	?	9–10	10	
**PrO**	1	1	**0**	1	1	?	1	**0**	1	1–2	
**PtO**	2	2	2–3	2	2	?	2	2	1–2	2	
**Loreal**	1	1	1	1	1	?	1	1	1	1	
**L-E**	no	yes	yes	no	no	?	no	yes	no	no	
**aTMP**	2 (rarely 1)	2	1	2	2	?	2–3	2	2–3	2	
**pTMP**	3 (rarely 2)	**2**	2	**2**	2–3	?	3	2–3	3–4	**2**	
**V**	**153**–**178**	**225**–**244**	190–230	**201**–**203**	**222**–**232**	**218**–**237**	176–206	**224**	179–207	**213**	
**SC**	**42**–**66**	**93**–**111**	60–91	**68**–**69**	**56**	61–102	**64**–**76**	**86**	**45**–**75**	**85**	
**PrC**	**divided**	entire	entire /divided	entire	**divided**	**divided**	entire	entire	**divided**	entire	
**BSC**	smooth	**keeled**	**feebly keeled**	**the 6–7 medial rows keeled**	smooth	?	smooth	**keeled**	smooth	**weakly keeled**	

### Phylogenetic analyses

For molecular analysis, a total of 20 samples was used, encompassing 18 samples from eight known *Lycodon* species (one sample of *L. fasciatus*, two samples of *L. flavozonatus*, four samples of *L. futsingensis*, two samples of *L. liuchengchaoi*, one sample of *L. multizonatus*, two samples of *L. rufozonatus*, four samples of *L. ruhstrati*, and two samples of *L. subcinctus*) and two samples of the unnamed species. Tissue samples were taken prior to fixation, and preserved in 99 % alcohol and stored at -40 °C.

Genomic DNA was extracted from muscle or liver tissue samples, using a DNA extraction kit from Tiangen Biotech (Beijing) Co., Ltd. A fragment of the mitochondrial cytochrome *b* (CYTB) gene was amplified using the primer pair L14910 (5’–GACCTGTGATMTGAAAACCAYCGTTGT-3’) and H16064 (5’– CTTTGGTTTACAAGAACAATGCTTTA-3’) following Burbrink et al. (2000). PCR amplification was run using the following cycling conditions: initial denaturing step at 94 °C for 5 min; followed by 35 cycles of 94 °C for 30 s, 48 °C for 1 min and 72 °C for 70 s; and final extension step at 72 °C for 10 min. PCR products were purified with spin columns and then sequenced with forward primers using BigDye Terminator Cycle Sequencing Kit as per the guidelines on an ABI Prism 3730 automated DNA sequencer by Guangzhou Tianyi Huiyuan Bio-tech Co., Ltd.

Twenty sequences from 12 known *Lycodon* species and two out-group sequences *Boiga cynodon* (Boie, 1872) and *Dasypeltis atra* Sternfeld, 1912, following Janssen et al. (2019) were obtained from GenBank and incorporated into our dataset (Table 2). DNA sequences were aligned by the Clustal W algorithm with default parameters (Thompson et al. 1997) and trimmed with gaps partially deleted in MEGA 6 (Tamura et al. 2013). The aligned dataset was tested in jmodeltest v2.1.2 (Darriba et al. 2012) with Akaike and Bayesian information criteria, all resulting the best-fitting nucleotide substitution models of GTR+I+G. Sequence data was analyzed using Bayesian inference (BI) in MrBayes 3.2.4 (Ronquist et al. 2012), and maximum likelihood (ML) in RaxmlGUI 1.3 (Silvestro and Michalak 2012). In the BI analysis, three independent runs were conducted, each being run for 2 million generations and sampled every 1000 generations with the first 25% samples were discarded as burn-in. In the ML analysis, the bootstrap consensus tree was inferred from 1000 replicates. Pairwise distances (p-distance) were calculated in MEGA6 using the uncorrected *p*-distance model.

**Table 2.** Localities, voucher information, and GenBank numbers for all samples used in this study.

*Lycodon* species	Voucher No.	Collection locality	GenBank No.	References
(1) *Lycodon cathaya* sp. nov.	SYS r001542	**China**: Huaping National NR, Longsheng County, Guangxi	MT602075	This study
(2) *Lycodon cathaya* sp. nov.	SYS r001630	**China**: Huaping National NR, Longsheng County, Guangxi	MT602076	This study
(3) *L. banksi*	VNUF R.2015.20	**Laos**: Khammouane Province	MH669272	Luu et al. 2018
(4) *L. butleri*	LSUHC:8365	**Malaysia**: Bukit Larut, Perak	KJ607892	Grismer et al. 2014
(5) *L. butleri*	LSUHC:9137	**Malaysia**: Bukit Larut, Perak	KJ607891	Grismer et al. 2014
(6) *L. cavernicolus*	LSUHC 9985	**Malaysia**: Perlis	KJ607889	Grismer et al. 2014
(7) *L. cavernicolus*	LSUHC 10500	**Malaysia**: Perlis	KJ607890	Grismer et al. 2014
(8) *L. fasciatus*	CAS 234875	**Myanmar**: Chin State	KC010365	Siler et al. 2013
(9) *L. fasciatus*	CAS 234957	**Myanmar**: Chin State	KC010366	Siler et al. 2013
(10) *L. fasciatus*	SYS r002401	**China**: Ruili City, Yunnan	MT625862	This study
(11) *L. flavozonatus*	SYS r001357	**China**: Bamianshan National NR, Guidong County, Hunan	MT625850	This study
(12) *L. flavozonatus*	SYS r001358	**China**: Bamianshan National NR, Guidong County, Hunan	MT625851	This study
(13) *L. futsingensis*	SYS r001250	**China**: Mt. Nankun, Huizhou City, Guangdong	MT625847	This study
(14) *L. futsingensis*	SYS r001494	**China**: Shimentai National NR, Yingde City, Guangdong	MT625853	This study
(15) *L. futsingensis*	SYS r001667	**China**: Gaoping Provincial NR, Renhua County, Guangdong	MT625857	This study
(16) *L. futsingensis*	SYS r002123	**China**: Gaoping Provincial NR, Renhua County, Guangdong	MT625861	This study
(17) *L. gongshan*	GP 3516	**China**: Lincang City, Yunnan	KP901022	Guo et al. 2015
(18) *L. gongshan*	GP 3546	**China**: Lincang City, Yunnan	KP901024	Guo et al. 2015
(19) *L. laoensis*	FMNH 258659	**Laos**: Salavan Province	KC010368	Siler et al. 2013
(20) *L. laoensis*	LSUHC 8481	**Cambodia**: Pursat Province	KC010370	Siler et al. 2013
(21) *L. liuchengchaoi*	SYS r001654	**China**: Shennongjia National NR, Hubei	MT625855	This study
(22) *L. liuchengchaoi*	SYS r001655	**China**: Shennongjia National NR, Hubei	MT625856	This study
(23) *L. namdongensis*	VNUF R.2017.23	**Vietnam**: Nam Dong Nature Reserve, Thanh Hoa	MK585007	Luu et al. 2019
(24) *L. meridionalis*	VNUF R.2017.54	**Vietnam**: Ninh Binh	MH669268	Luu et al. 2018
(25) *L. meridionalis*	VNUF R.2017.88	**Vietnam**: Ninh Binh	MH669269	Luu et al. 2018
(26) *L. multizonatus*	KIZ01623	**China**: Luding County, Sichuan	KF732926	Lei et al. 2014
(27) *L. multizonatus*	SYS r002411	**China**: Baishuijiang National NR, Longnan City, Gansu	MT625863	This study
(28) *L. pictus*	ZFMK93746	**Vietnam**: Ha Lang District, Cao Bang	MN395829	Janssen et al. 2019
(29) *L. pictus*	ZFMK93747	**Vietnam**: Ha Lang District, Cao Bang	MN395830	Janssen et al. 2019
(30) *L. rufozonatus*	SYS r001770	**China**: Mt. Tiantai, Zhejiang	MT625858	This study
(31) *L. rufozonatus*	SYS r002061	**China**: Yangjifeng National NR, Guixi City, Jiangxi	MT625860	This study
(32) *L. ruhstrati*	SYS r001275	**China**: Shaowu Jiangshi Provincial NR, Nanping City, Fujian	MT625848	This study
(33) *L. ruhstrati*	SYS r001309	**China**: Jiulianshan National NR, Longnan County, Jiangxi	MT625849	This study
(34) *L. ruhstrati*	SYS r001362	**China**: Bamianshan National NR, Guidong County, Hunan	MT625852	This study
(35) *L. ruhstrati*	SYS r001631	**China**: Huaping National NR, Longsheng County, Guangxi	MT625854	This study
(36) *L. semicarinatus*	N/A	**Japan**: Ryukyu Archipelago	AB008539	Kumazawa et al. 1996
(37) *L. subcinctus*	SYS r001155	**China**: Neilingding Island, Shenzhen City, Guangdong	MT625846	This study
(38) *L. subcinctus*	SYS r001943	**China**: Shimentai National NR, Yingde City, Guangdong	MT625859	This study
(39) *L. synaptor*	GP 3515	**China**: Lincang City, Yunnan	KP901021	Guo et al. 2015
(40) *L. synaptor*	GP 3545	**China**: Lincang City, Yunnan	KP901023	Guo et al. 2015
**Outgroups**				
(41) *Boiga cynodon*	KU 324614	**Philippines**: Negros Occidental	KC010340	Siler et al. 2013
(42) *Dasypeltis atra*	CAS 201641	**Uganda**: Kabale district	AF471065	Lawson et al. 2005

## Results

**Figure 2.**
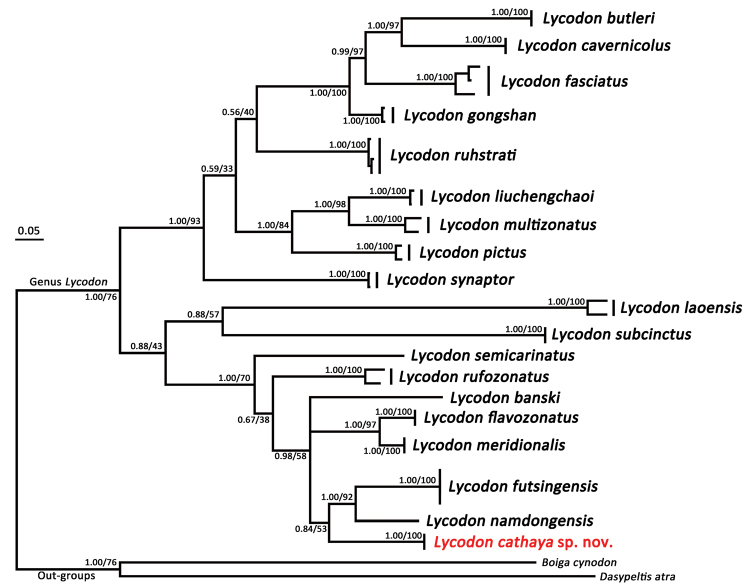
Bayesian Inference and Maximum Likelihood phylogenies.

The CYTB nucleotide sequence matrix contained 1050 characters without insertion deletions. The MP and BI analyses produced essentially identical topologies, which were integrated in Fig. 2. Major nodes of the tree were supported with the Bayesian posterior probabilities (BPP) > 0.95 and the bootstrap supports (BS) for Maximum Likelihood analysis > 75. Uncorrected *p*-distances among *Lycodon* species based on the CYTB gene are shown in Table 3.

**Table 3.** Uncorrected *p*-distances among *Lycodon* species based on partial mitochondrial CYTB gene.

ID	*Lycodon* species	1–2	3	4–5	6–7	8–10	11–12	13–16	17–18	19–20	21–22	23	24–25	26–27	28–29	30–31	32–35	36	37–38	39–40
**1–2**	*Lycodon cathaya* sp. nov.	0																		
**3**	*L. banksi*	9.6	–																	
**4–5**	*L. butleri*	17.3	20.2	0																
**6–7**	*L. cavernicolus*	17	18.7	9.6	0															
**8–10**	*L. fasciatus*	12–13.7	14.6–16.3	10.4–11.5	9.8–10.7	0.7–1.8														
**11–12**	*L. flavozonatus*	9.3	10.1	18	17.4	14.2–14.6	0													
**13–16**	*L. futsingensis*	8.9	9.5	16.9	17.1	14.8–15.4	9.2	0												
**17–1**8	*L. gongshan*	14.5–14.7	14.9–15.1	8.9–9.1	7.6–7.7	7.1–8.5	14.1–14.3	14.1–14.3	0.1											
**19–20**	*L. laoensis*	16.6–17.4	17–17.2	20.1–21.3	17.6–19.2	16.3–17.9	16.6–17.2	17.8–18.8	15.4–17	0.2										
**21–22**	*L. liuchengchaoi*	16.3–16.5	17–17.2	13.7–13.8	13.4–13.5	12.3–13	16.3–16.5	14.6–14.8	10.1–10.5	18.7–20.1	0.1									
**23**	*L. namdongensis*	7.9	8.8	17.1	16.5	14.2–15.2	8	6.8	14.3–14.5	17.2–18.2	15.7–15.9	–								
**24–25**	*L. meridionalis*	7.9	9.6	17.2	17.4	13.1–13.6	2.7	8.5	13.7–13.9	15.9–16.5	15.3–15.5	8.1	0							
**26–27**	*L. multizonatus*	14.8–15.1	16–16.7	14.2–14.5	15.6–15.8	12.7–13	16.1–16.5	14.6–15.4	11.9–12.1	18–19.4	6.7–7.1	15.2–15.9	15.3	1.6						
**28–29**	*L. pictus*	14.3–14.7	15.7–15.9	14.2–14.8	15.3–16	12.8–13.6	13.8–14.2	14.9	12–12.5	17.6–18.6	9.5–9.7	14.1–14.5	14	10–10.4	0.6					
**30–31**	*L. rufozonatus*	10.7–11.2	12.2–12.7	17.1	17.9–18.6	15.2–15.9	8.9–9.4	10.1–11	14.5–14.8	17.7–18.7	15.9–16.7	10.4–10.6	9.4–9.6	15.2–15.7	14–15.1	2				
**32–35**	*L. ruhstrati*	14–14.4	15.9–16.3	13.4–13.6	12.9–13.1	12.2–13.2	13.8–14	15.1–15.3	9.7–10.3	16–17.6	11.5–11.9	14.9–15.3	13.4–13.6	10.6–11.1	10.8–11.4	14–14.6	0–0.3			
**36**	*L. semicarinatus*	11.2	12.2	17.7	18.9	15.2–15.5	11.8	12.8	15.1–15.3	18.1–18.3	16.4–16.6	12.3	11.2	15.6	15.9–16.1	10.5–10.9	15–15.4	–		
**37–38**	*L. subcinctus*	15.8	17.3	18.4	16.5	16.2–16.8	15.5	16.2	16.1–16.3	15.7–16.5	17.9–18.1	16.1	15.9	17.9–18.1	16.1–17.1	16.4–17	13.6–13.8	17.9	0–0.3	
**39–40**	*L. synaptor*	16.6	18	15.4	13	12.8–13	15.3	15.4	11.5–11.6	18.9–19.5	14–14.2	15.4	15.1	14.3–14.7	12.2–12.4	14.1–14.5	11.1–11.2	17.1–17.6	17.6	0

The phylogenetic topologies are very similar to those recovered by previous study (Janssen et al. 2019). The unnamed *Lycodon* samples from Guangxi, southern China clustered in a monophyletic lineage with high nodal supports (BPP = 1.00 and = BS 100). This lineage are genetically differentiated from all congeners with the uncorrected *p*-distance ≥ 7.9%, which is significant when compared with that between other recognized species (e.g., *p*-distance = 2.7 % between *L. flavozonatus* and *L. meridionalis*, *p*-distance 6.8 % between *L. futsingensis* and *L. namdongensis*, and *p*-distance = 6.7–7.1 % between *L. liuchengchaoi* and *L. multizonatus*). The phylogenetic placement of the new lineage is largely unresolved, even though it forms the sister taxon to the clade composed of *L. futsingensis* and *L. namdongensis* while the nodal support is insignificant.

Moreover, it is noteworthy that the unnamed *Lycodon* possesses significant morphological differences that can be easily distinguished from all other congeners (see below). Therefore, based on the combination of molecular and morphological data, we describe the unnamed population from Huaping Nature Reserve, Guangxi, southern China as a new species, *Lycodon cathaya* sp. nov.

### Taxonomic account

#### Lycodon cathaya sp. nov.

Taxon classificationAnimaliaSquamataColubridae

A220BD6D-D8BE-5F4B-BE8D-A8E458ED8A5E

http://zoobank.org/BA36B7DE-36BD-4B3C-A317-BF4B8E451A26

**Figure 3.**
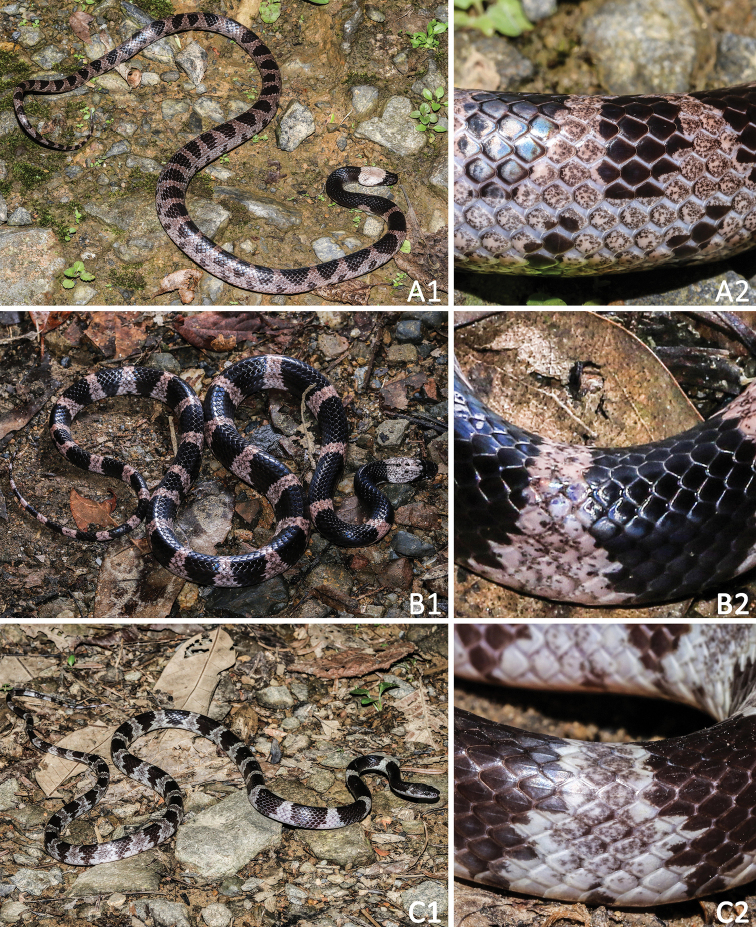
General aspects in life and close-ups of body scales of **A***Lycodon cathaya* sp. nov. (SYS r001542, holotype) from Huaping Nature Reserve, Guangxi, China **B***L. futsingensis* (SYS r002123) from Gaoping Nature Reserve, Shaoguan City, Guangdong, China, and **C***L. ruhstrati* (SYS r001631) from Huaping Nature Reserve, Guangxi, China.

**Figure 4.**
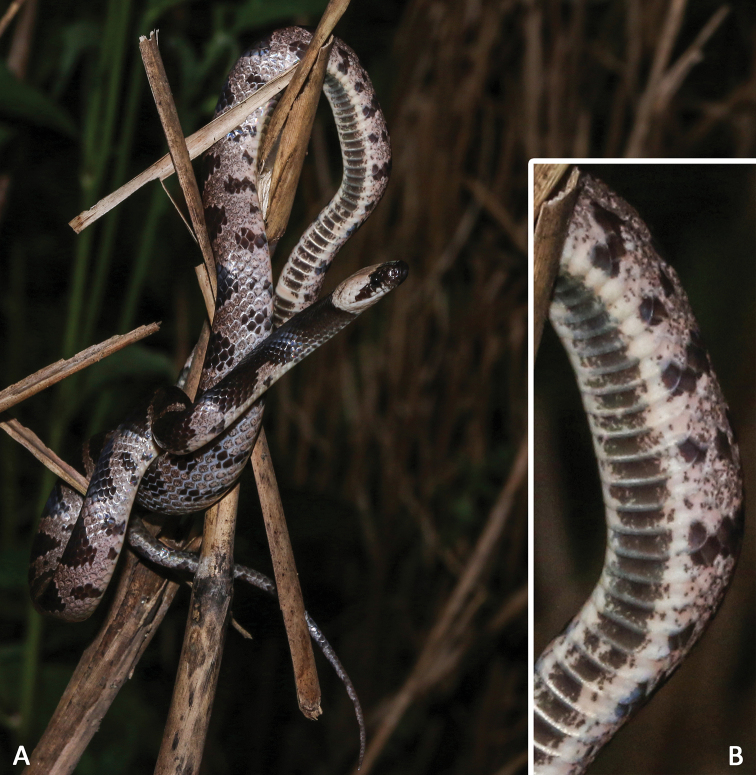
General aspect of *Lycodon cathaya* sp. nov. (SYS r001542, holotype) in life when observed.

**Figure 5.**
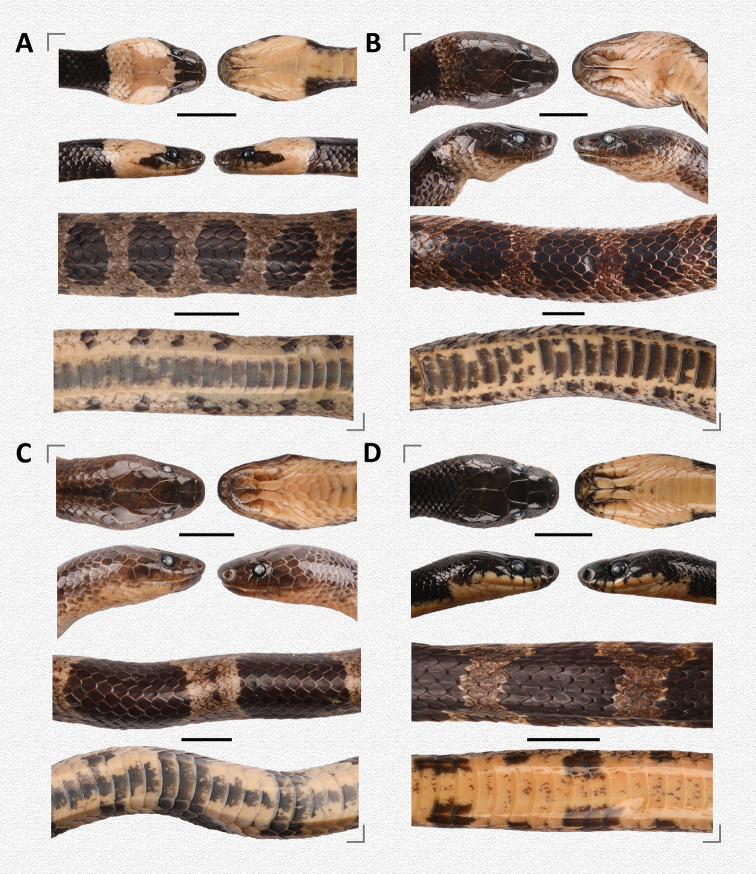
Comparative characters of head scalation and color patterns (in preservative) of **A***Lycodon cathaya* sp. nov. (SYS r001542, holotype) **B***Lycodon cathaya* sp. nov. (SYS r001630, paratype) **C***L. futsingensis* (SYS r002123), and **D***L. ruhstrati* (SYS r001631). Scale bars: 10 mm.

Figures 3A , 4 , 5A, B

##### Holotype.

SYS r001542, adult male, collected on 20 July 2016 by Jian Wang from Huaping Nature Reserve (25.62521N, 109.91376E (DD); ca 1000 m a.s.l.), Longsheng County, Guilin City, Guangxi Zhuang Autonomous Region, China.

##### Paratypes.

SYS r001630, adult male, collected on 2 September 2016 by Jian Wang from Huaping Nature Reserve (25.62667N, 109.91351E (DD); ca 850 m a.s.l.).

##### Etymology.

The specific name *cathaya* is a noun referring to the monotypic botanic genus *Cathaya* Chun & Kuang, 1958. The single species *C. argyrophylla* Chun & Kuang, 1958 is an endangered relict plant, and was firstly discovered from Huaping Nature Reserve by the investigation team of Sun Yat-sen University. In memory of the predecessors and their contributions on the taxonomy of Chinese flora and fauna, we denominate this new snake species from Huaping Nature Reserve as *Lycodon cathaya* sp. nov. Its common name is suggested as “Huaping wolf snake” in English and “Hua Ping Bai Huan She (花坪白环蛇)” in Chinese.

##### Diagnosis.

*Lycodon cathaya* sp. nov. can be differentiated from its congeners by the combination of the following morphological characters: (1) dorsal scales in 17–17–15 rows, smooth throughout; (2) supralabials eight, third to fifth in contact with eye, infralabials 9; (3) ventral scales 199–200 (plus two preventral scales), subcaudals 78; (4) loreal single, elongated, in contact with eye or not, not in contact with internasals; (5) a single preocular not in contact with frontal, supraocular in contact with prefrontal, two postoculars; (6) maxillary teeth 10 (4+2+2+2); (7) two anterior temporals, three posterior temporals; (8) precloacal plate entire; (9) ground color from head to tail brownish black, with 31–35 dusty rose bands on body trunk, 13–16 on tail; (10) bands in 1–2 vertebral scales broad in minimum width; (11) bands separate ground color into brownish black ellipse patches, similar arrangement in a row along the top of body and tail; (12) elliptical patches in 3–6 scales of the vertebral row in maximum width; (13) ventral surface of body with a wide brownish black strip, margined with a pair of continuous narrow greyish white ventrolateral lines.

##### Comparisons.

The detailed comparisons among all *Lycodon* congeners are given in Table 1, with distinguishing characters marked in bold.

In our phylogenetic tree (Fig. 2), *Lycodon cathaya* sp. nov. (Figs 3A, 4, 5A, B) is relatively close to *L. futsingensis* (Figs 3B, 5C) and *L. namdongensis*. However, the new species possesses significant morphological differences: (1) 10 maxillary teeth (vs. MT 12–15 in *L. futsingensis*), bands on dorsal body and tail link with each other and separate ground color into ellipse patches (vs. bands on dorsal body and tail separate with each other in *L. futsingensis*), venter line on ventral body margined with a pair of continuous ventrolateral line (vs. ventrolateral lines discontinuous, interrupted by black patches in *L. futsingensis*); (2) ten maxillary teeth (vs. MT 12 in *L. namdongensis*), nine infralabials (vs. IFL ten in *L. namdongensis*), two postocular (vs. PtO 3 in *L. namdongensis*), three posterior temporals (vs. pTMP 3 in *L. namdongensis*), ventral scales 199–200 (vs. V 218 in *L. namdongensis*), dorsal body with 31–35 dusty rose bands (vs. dorsal body with 23 greyish cream bands in *L. namdongensis*).

*Lycodon cathaya* sp. nov. can be further distinguished from *L. ruhstrati* (Figs 3C, 5D), which used to be confused with *L. futsingensis*, to which it is morphologically similar (Pope 1935; Vogel et al. 2009), by the following morphological characters: (1) dorsal scales smooth throughout (vs. dorsum with keeled scales); (2) subcaudals 78 (vs. subcaudals ≥ 90); (3) bands on dorsal body and tail link with each other and separate ground color into ellipse patches (vs. bands on dorsal body and tail separate with each other); (4) ventral with a brownish black venter strip margined with a pair of continuous greyish white ventrolateral lines (vs. brownish black venter strip absent, and ventrolateral lines discontinuous, interrupted by black patches).

*Lycodon cathaya* sp. nov. can be significantly distinguished from *L. albofuscus*, *L. banksi*, *L. butleri*, *L. capucinus*, *L. cardamomensis*, *L. carinatus*, *L. cavernicolus*, *L. davidi*, *L. fasciatus*, *L. flavozonatus*, *L. gammiei*, *L. gibsonae*, *L. gongshan*, *L. gracilis*, *L. liuchengchaoi*, *L. meridionalis*, *L. multifasciatus*, *L. nympha*, *L. orientalis*, *L. paucifasciatus*, *L. rosozonatus*, *L. semicarinatus*, *L. septentrionalis*, *L. sidiki*, *L. subannulatus*, *L. subcinctus*, *L. synaptor*, *L. tristrigatus* and *L. zoosvictoriae* by its smooth dorsal scales (vs. dorsal body with keeled scales). By having dorsal scales in 17–17–15 rows, *Lycodon cathaya* sp. nov. can be easily distinguished from *L. alcalai* (DSR 19–17–15), *L. bibonius* (DSR 19–17–15), *L. chrysoprateros* (DSR 19–17–15), *L. davisonii* (DSR ?–13–?), *L. dumerilii* (DSR 19–17–15), *L. fausti* (DSR 19–17–15), *L. ferroni* (DSR ?–13–?), *L. kundui* (DSR 15–15–15), *L. muelleri* (DSR 19–17–15), *L. philippinus* (DSR ?–15–?), *L. solivagus* (DSR 19–17–15) and *L. stormi* (DSR ?–19–?). From the remaining 18 congeners, *Lycodon cathaya* sp. nov. can be easily distinguished from *L. ophiophagus*, *L. pictus*, and *L. zawi* by having fewer maxillary teeth; from *L. anamallensis*, *L. effraenis*, *L. flavicollis*, *L. flavomaculatus*, *L. hypsirhinoides*, *L. laoensis*, *L. striatus*, and *L. travancoricus* by having fewer supralabials; from *L. anamallensis*, *L. aulicus*, *L. effraenis*, *L. flavicollis*, *L. flavomaculatus*, *L. hypsirhinoides*, *L. laoensis*, *L. multizonatus*, *L. ophiophagus*, *L. pictus*, and *L. striatus* by having fewer infralabials and from *L. mackinnoni* by having more infralabials; from *L. aulicus*, *L. flavicollis*, *L. flavomaculatus*, *L. hypsirhinoides*, *L. jara*, *L. laoensis*, *L. mackinnoni*, *L. multizonatus*, *L. sealei*, *L. striatus*, *L. tessellatus*, and *L. tiwarii* by having an entire precloacal plate (vs. precloacal plate divided); from *L. jara*, *L. mackinnoni*, and *L. striatus* by having more ventrals and from *L. pictus*, *L. tessellatus*, and *L. tiwarii* by having fewer ventrals; from *L. anamallensis*, *L. flavicollis*, *L. hypsirhinoides*, *L. jara*, *L. laoensis*, *L. flavomaculatus*, *L. mackinnoni*, *L. multizonatus*, *L. striatus*, *L. tessellatus* and *L. zawi* by having more subcaudals and from *L. ophiophagus* and *L. pictus* by having fewer subcaudals; from *L. effraenis* and *L. sealei* by the presence of a single loreal (vs. loreal absent).

##### Description of holotype.

Adult male. Body slender, TL 562.5 mm (SVL 451.4 mm, TaL 111.1 mm, TaL/TL ratio 0.198); dorsal scales in 17–17–15 rows, smooth throughout, the vertebral scales not enlarged; head elongate, moderately distinct from neck, rather flattened, longer than wide, and narrow anteriorly, HL 17.2 mm, HW 11.1 mm (HW/HL ratio 0.643); eye large, ED 2.2 mm, pupil vertically elliptic; rostral triangular, much broader than high, barely visible from above; nostril lateral, located in the middle of nasal; nasal divided into two scales by nostril; two internasals, anteriorly rounded, almost as wide as high, bordered by two large, pentagonal prefrontals posteriorly; a single enlarged hexagonal frontal, narrowed posteriorly; parietals paired, longer than wide, in contact with each other medially, with upper anterior and posterior temporals, paraparietal laterally and four nuchal scales posteriorly; paraparietal slightly elongate, nearly rectangular; one elongated loreal on each side, in contact with eye, not in contact with internasals; one preocular located above loreal, in contact with eye and supraocular posteriorly, with prefrontal anteriorly, and not in contact with frontal; two postoculars, almost equal in length, upper one in contact with eye anteriorly, with supraocular and parietal, and with upper temporal posteriorly, lower one in contact with eye anteriorly, with anterior temporals posteriorly, and with fifth and sixth supralabials below; eight supralabials on each side, first and second in contact with nasal, third to fifth entering orbit; nine infralabials on each side, first pair in broad contact with each other, first to fourth in contact with anterior pair of chin shields, fourth to fifth in contact with posterior chin shields; two pairs of chin shields, elongate, anterior pair larger, second pair meeting in midline; two anterior temporals, almost equal in size, three posterior temporals, upper one smallest, lower one largest; 199 ventrals plus two preventrals; 78 pairs of subcaudals, excluding tail tip; precloacal plate entire.

##### Dentition.

10 (4+2+2+2) maxillary teeth on both sides, four small anterior teeth, enlarged posteriorly; two noticeably enlarged snag shaped teeth (second largest); two moderately enlarged teeth; two moderately enlarged kukri liked teeth (the anterior one larger, both with posterior cutting edges). Diastemas present between the above-mentioned maxillary teeth groups.

##### Hemipenis.

Hemipenis elongated, apex not fully everted after injection of formalin. Truncus bulbous, lower 1/3 smooth without spines, spine ornamentation starting at upper part with somewhat enlarged, medium sized spines. Apex with dense microspines. Sulcus spermaticus stretches to base of apex. Apex not fully everted, ending somewhat widened with an oblique opening, with microspines inside.

##### Coloration of holotype.

In life (Figs 3A, 4), dorsal surface of head brownish black, a distinctly dusty rose collar band that crosses over the head and nape of the neck; ventral surface of head almost white, mental, the 1^st^–3^rd^ supralabials and the anterior pair of chin shields with brownish black patches, the 4^th^ and 5^th^ and the posterior pair of chin shields with brownish black mottles. Ground color of dorsal surface brownish black, with 35 transverse dusty rose bands on body trunk and 16 similarly colored bands on tail, including two incomplete bands between collar band and the first complete transverse band; each band in 1–2 scales of the vertebral row in minimum width and widen laterally to a width of 3–4 scales; bands link with each other in ventrolateral body and tail, and separate the ground color into brownish black ellipse patches: such patches in 3–6 scales of the vertebral row in maximum width, and arranged in a row along the top of body and tail; a brownish black ventrolateral blotch on each ventrolateral side of bands. Middle of each ventral with irregular brownish black blotches forming a relatively continuous venter strip, and greyish white on both sides, forming a pair of continuous ventrolateral lines, which run in parallel along the venter strip. Subcaudals almost entirely light brown. In preservative (Fig. 5A), the collar band faded to beige, bands become darker, and the ventral surface faded to beige.

##### Variations.

Measurements, body proportions and scale counts of the two specimens are listed in Table 4. The paratype has a relatively small and faint collar band, just crossing over the nape of the neck; dorsal bands are faint and there are more dark brown speckles than in the holotype. It appears that this specimen represents an older age group than the holotype, and differences in coloration may indicate an ontogenetic development. The loreal is in contact with eye in the holotype, while the loreal is separated from the eye by the preocular and the third supralabial.

**Table 4.** Measurements, scale counts, and body proportions of *Lycodon cathaya* sp. nov.

	Voucher number	
Character	1542	1630
**Age**	adult	adult
**Sex**	male	male
**SVL**	451.4	730.1
**TaL**	111.1	180.5
**TL**	562.5	910.6
**TaL/TL**	0.198	0.198
**HL**	17.2	23.3
**HW**	11.1	14.6
**HW/HL**	0.643	0.627
**ED**	2.2	3.0
**DSR**	17–17–15	17–17–15
**SpL**	8	8
**IfL**	9	9
**IFL-1CS**	1^st^–4^th^	1^st^–4^th^
**IFL-2CS**	4^th^–5^th^	4^th^–5^th^
**CS**	2	2
**V**	199	200
**Sc**	78	78
**S-V Bands**	35	31
**TaL Bands**	16	13
**MT**	10	10

##### Distribution and habits.

Currently, *Lycodon cathaya* sp. nov. is only known from its type locality, Huaping Nature Reserve (Fig. 1; ca 850–1000 m a.s.l.), and is sympatric with *L. meridionalis* and *L. ruhstrati*. All of them are nocturnal species. The holotype was observed climbing on a wilted bush by the roadside, approximately half a meter above the ground (Fig. 4). The paratype and an individual of its sympatric species *L. ruhstrati* is (Fig. 3C) were found on the ground on the same night. The surrounding environment consisted of well-preserved montane evergreen broad-leaved forest or mixed forest.

## Discussion

The description of *Lycodon cathaya* brings the total species number of this genus to 64, 16 of which occur in China. The new discovery further emphasizes the very high diversity level of the genus *Lycodon* (Zhao 1981; Zhao et al. 1998; Zhao 2006; Luo et al. 2010).

The Huaping Nature Reserve is located in the hilly region among Guangxi, Hunan, and Guizhou. Thus, the new species is expected to occur in southwestern Hunan and southeastern Guizhou. The area within the jurisdiction of Huaping Nature Reserve has been well valued and protected by relevant local departments, with a considerable amount of research and investigation efforts having been conducted. However, further research on the true distribution, population sizes and trends, habitat conditions and conservation actions are urgently needed in the potential distribution areas outside the jurisdiction of Huaping Nature Reserve. Moreover, since the rapid and notable developments on the knowledge about the Chinese herpetofauna, the hilly regions in southern China have received more attention and a number of new species have been discovered in the recent years (Chen et al. 2018; Li et al. 2018; Lyu et al. 2018; Peng et al. 2018; Sung et al. 2018; Wang et al. 2018ab; Chen et al. 2019; Lyu et al. 2019ab; Wang et al. 2019abc; Wang et al. 2020ab); this in turn strengthens appeals for more powerful and targeted conservation actions in these regions.

## Supplementary Material

XML Treatment for Lycodon cathaya

## Appendix I

**Examined specimens**

***Lycodon flavozonatus*** (N = 11): Nanling Nature Reserve, Guangdong, China: SYS r000819; Mt. Jinggang, Jiangxi, China: SYS r000317, 001956, 001972; Mt. Huanggang, Jiangxi, China: SYS r000640; Mt. Bamian, Hunan, China: SYS r001357, 001358, 001360, 001778; Mt. Wuyi, Fujian, China: SYS r001722; Mt. Dongbai, Zhejiang, China: SYS r001772.

***Lycodon futsingensis*** (N = 9): Nanling Nature Reserve, Guangdong, China: SYS r000051, 000054; Mt. Wutong, Shenzhen, Guangdong, China: SYS r000617, 001016; Gaoping Nature Reserve, Shaoguan, China: SYS r001542, 001630, 001667, 002123; Shimentai Nature Reserve, Guangdong, China: SYS r001494.

***Lycodon liuchengchaoi*** (N = 1): Shimentai Nature Reserve, Guangdong, China: SYS r002114.

***Lycodon meridionalis*** (N = 5): Heishiding Nature Reserve, Guangdong, China: SYS r001355, 002053; Mt. Jiuwan, Guangxi, China: SYS r001812; Mt. Dayao, Guangxi, China: SYS r002326, 002327.

***Lycodon rosozonatus*** (N = 2): Jianfengling, Hainan, China: SYS r001617; Bawangling, Hainan, China: SYS r002164.

***Lycodon rufozonatus*** (N = 3): Mt. Jinggang, Jiangxi, China: SYS r000318; Mt. Bamian, Hunan, China: SYS r001361; Mt. Tiantai, Zhejiang, China: SYS r001770.

***Lycodon ruhstrati*** (N = 7): Mt. Jiulian, Jiangxi, China: SYS r001309; Jiangshi Nature Reserve, Fujian, China: SYS r001275; Mt. Jinggang, Jiangxi, China: SYS r001256; Mt. Qiyun, Jiangxi, China: SYS r000882; Mt. Bamian, Hunan, China: SYS r001362; Huaping Nature Reserve, Guangxi, China: SYS r001631, 001633.

***Lycodon subcinctus*** (N = 13): Sun Yet-sen University, Zhuhai, Guangdong, China: SYS r001013; Heishiding Nature Reserve, Guangdong, China: SYS r001523, 001757; Neilingding Island, Shenzhen, Guangdong, China: SYS r001155, 001511; Tiegang Reservoir, Shenzhen, Guangdong, China: SYS r001430; Maluanshan Country Park, Shenzhen, Guangdong, China: SYS r002146; Shimentai Nature Reserve, Guangdong, China: SYS r001943, 002021; Mt. Diaoluo, Hainan, China: SYS r001621; Xishuangbanna, Yunnan, China: SYS r000689, 000690.
